# “It wasn’t as bad as I thought it would be”: a qualitative study of early stage non-small cell lung cancer patients after treatment

**DOI:** 10.1186/s13104-017-2956-3

**Published:** 2017-11-29

**Authors:** Sara E. Golden, Charles R. Thomas, Mark E. Deffebach, Mithran S. Sukumar, Paul H. Schipper, Brandon H. Tieu, Andrew Y. Kee, Andrew C. Tsen, Christopher G. Slatore, For the Early Stage Lung Cancer Comparative Effectiveness Research Consortium, Mark E. Deffebach, Mithran S. Sukumar, Paul H. Schipper, Brandon H. Tieu, Charles R. Thomas, Charlotte D. Kubicky, John M. Holland, Andrew Y. Kee, Andrew C. Tsen, John R. Handy, Steven Seung, Michael A. Myers, Dennis L. Febinger, Timur Mitin, Srinivas R. Mummadi, Kelli D. Salter, David G. Tse, Thomas D. Wynne

**Affiliations:** 1 Health Services Research & Development, VA Portland Health Care System, 3710 SW US Veterans Hospital Rd., R&D 66, Portland, OR 97239, USA; 2 Department of Radiation Medicine, Oregon Health & Science University, Portland, OR USA; 3 Department of Medicine, Oregon Health & Science University, Portland, OR USA; 4 Section of Pulmonary & Critical Care Medicine, VA Portland Health Care System, Portland, OR USA; 5 Department of Surgery, Oregon Health & Science University, Portland, OR USA; 6 Division of Radiation Oncology, Legacy Health System, Portland, OR USA; 7 Division of Cardiothoracic Surgery, Legacy Health System, Portland, OR USA

**Keywords:** Thoracic diseases, Patient-centered outcomes, Communication

## Abstract

**Objective:**

While surgical resection is recommended for most patients with early stage lung cancer, stereotactic body radiotherapy (SBRT) is being increasingly utilized. Provider-patient communication regarding risks/benefits of each approach may be a modifiable factor leading to improved patient-centered outcomes. Our objective was to determine a framework and recommended strategies on how to best communicate with patients with early stage non-small cell lung cancer (NSCLC) in the post-treatment setting. We qualitatively evaluated the experiences of 11 patients with early clinical stage NSCLC after treatment, with a focus on treatment experience, knowledge obtained, communication, and recommendations. We used conventional content analysis and a patient-centered communication theoretical model to guide our understanding.

**Results:**

Five patients received surgery and six received SBRT. Both treatments were generally well-tolerated. Few participants reported communication deficits around receiving follow-up information, although several had remaining questions about their treatment outcome (mainly those who underwent SBRT). They described feeling anxious regarding their first surveillance CT scan and clinician visit. Overall, participants remained satisfied with care because of implicit trust in their clinicians rather than explicit communication. Communication gaps remain but may be addressed by a trusting relationship with the clinician. Patients recommend clinicians give thorough explanations and personalize when possible.

## Introduction

With the advent of lung cancer screening, more people will be diagnosed with early stage disease [1]. Surgery is the recommended therapy for stage I non-small cell lung cancer (NSCLC) [2, 3] although stereotactic body radiotherapy (SBRT) is increasingly utilized. SBRT is typically performed in individuals who either refuse or cannot tolerate surgery [3, 4]. Nonetheless, SBRT may become an equivalent or potentially even better option since, in a limited trial, patients who were treated with SBRT had lower mortality and better global health-related quality of life (HR-QOL) 3.5 years after treatment than patients treated with surgery [5]. There are currently little data regarding how clinicians or patients make decisions about these two treatments for early stage lung cancer, and no data on decision making strategies that patients prefer to utilize. It is important to determine the best way to communicate with patients with early stage lung cancer about post-treatment procedures since communication may be one of only a few modifiable factors that could improve health outcomes.

To our knowledge, the current analysis represents the first prospective qualitative comparison and contrast assessment of the experience of patients who underwent either surgical or radiation treatment for early stage NSCLC, primarily focusing on patient-clinician communication strategies.

## Main text

### Methods

We qualitatively evaluated the experiences of patients with clinical stage I NSCLC before treatment, and then at 1 month and 1 year after treatment. The current analysis is based on the two follow-up interviews. We included patients treated at three medical centers in the northwest US: the Veterans Administration Portland Health Care System; Oregon Health & Science University (OHSU); and Legacy Health. We enrolled patients with suspected or confirmed stage I NSCLC being considered for curative treatment during 2014–2015. Pathologic confirmation of lung cancer was not an inclusion criterion [6]. We excluded patients with a history of lung cancer in the past 5 years, those who scored < 17/30 on the St. Louis University Mental Status Examination, had severe hearing impairment, were non-English speaking, lived in a skilled nursing facility, or were diagnosed with psychotic or cognitive disorders. The Joint Internal Review Board of the VA Portland Health Care System and OHSU (#10340), and the Legacy IRB, approved this study. All participants completed written informed consent. We completed recruitment after 13 participants, as we had reached thematic saturation at each study site [7, 8].

Our interview guide allowed for deviations as necessary and included questions about the treatment and post-treatment experience, gaps in knowledge, communication, and perceived barriers and facilitators to high quality care. Participants self-reported demographic and smoking characteristics. Using standardized report forms, we collected diagnosis and treatment information from the electronic medical record.

We used directed content analysis [9] and ATLAS.ti 7.1.7 (ATLAS.ti GmbH, Berlin, Germany) to organize and analyze the interviews. Each participant is identified by a randomly assigned letter not related to his or her name or treating hospital system, with a letter after a hyphen indicating if they were treated by SBRT (“R”) or surgery (“S”), then ending with a number to indicate if the reference came from the 1 month post-treatment interview (“2”) or the 1 year post-treatment interview (“3”). Individual participants are referred to as “she/her” to protect anonymity.

**Fig. 1:**
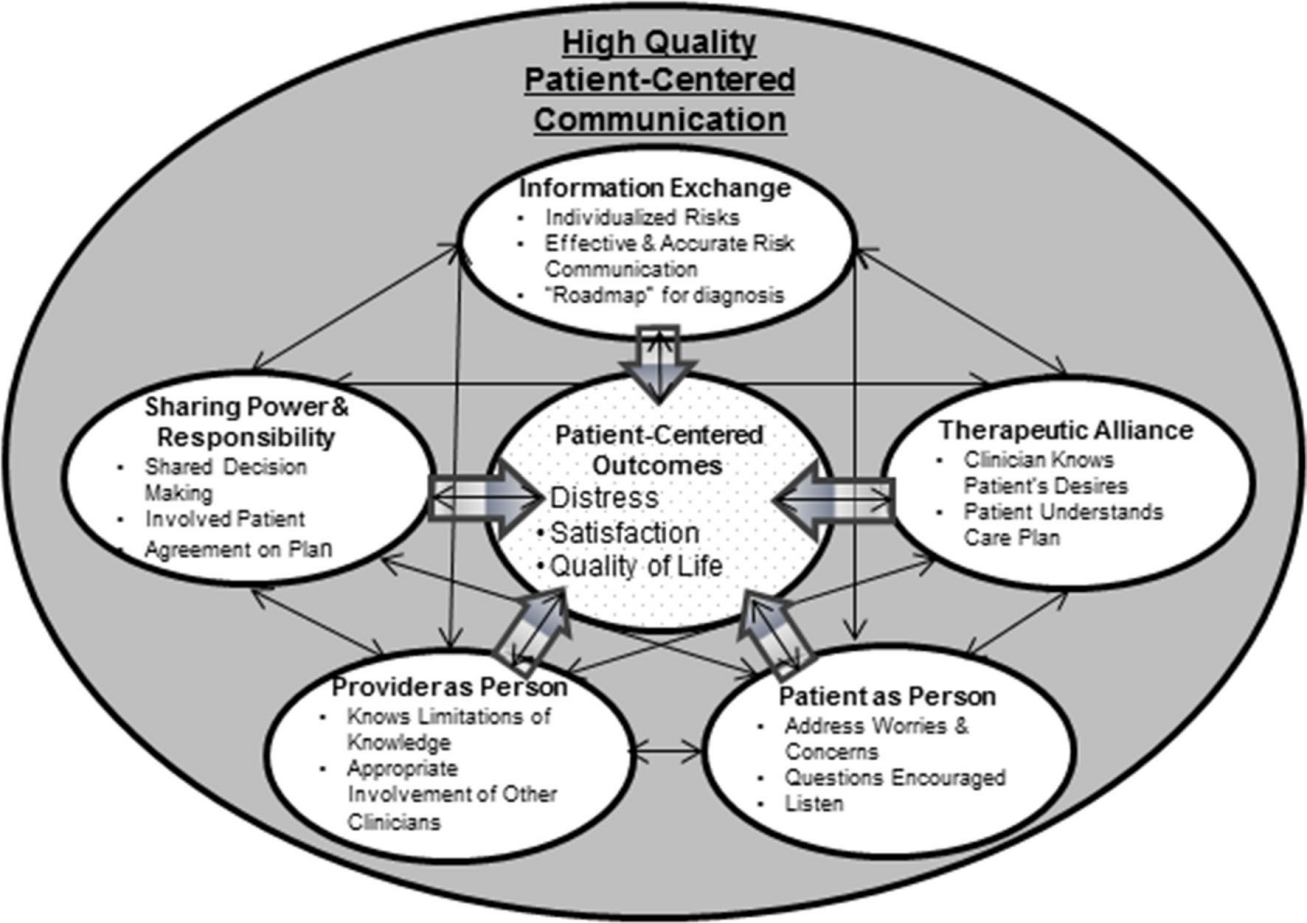
Patient-centered communication model

We used a patient-centered communication (PCC) theoretical model [10] to guide our understanding of the communication strategies, but the flexibility of the interview guide allowed other themes to emerge. The PCC domains include: information exchange; patient as person (consideration of patients’ feelings, preferences, and values); sharing power and responsibility (shared decision making); therapeutic alliance (the need for patient and provider to be “on the same page”); and clinician as person (Fig. 1) [11], although only the former three domains are discussed here since the latter two were not fully assessed during the interviews.

### Results

Of the initial 13 participants, 11 are discussed herein. Eleven completed the 1 month post-treatment (visit 2) interview, and 10 completed the 1 year post-treatment (visit 3) interview. Most participants were white (82%), former smokers (82%), and female (55%). Table 1 describes participant characteristics. We did not find substantially different themes based on care location.

**Table 1:** Self-reported subject characteristics, n = 11

Characteristic	N (%)^a^ or mean (SD)	SBRT only (n = 6) N (%) or mean (SD)	Surgery only (n = 7) N (%) or mean (SD)
Treatment location, n (%)			
VA Portland Health Care System	5 (45%)	2	3
Oregon Health & Science University	3 (27%)	1	2
Legacy Health System	3 (27%)	3	0
Age (year), mean (SD)	71 (10.5)	73 (13.6)	69 (6.0)
Male, n (%)	5 (45%)	2 (33%)	3 (43%)
Race/ethnicity, n (%)			
White	9 (82%)	5 (83%)	4 (57%)
Smoking status, n (%)			
Current smoker	2 (18%)	1 (17%)	1 (14%)
Former smoker	9 (82%)	5 (83%)	4 (57%)
Education, n (%)			
High school or less	5 (45%)	3 (50%)	2 (29%)
Employment status, n (%)			
Retired, disabled, or currently not working	9 (82%)	5 (83%)	4 (57%)
Income, n (%)			
$60,000 or more	5 (45%)	2 (33%)	3 (43%)

^a^Percents are of non-missing data. May not add up to 100% due to rounding

#### Patient as person

##### Treatment experience

Both treatments were generally well-tolerated and all participants were pleased with their treatment, care, and current QOL (Table 2). As participant M-R-2 said, “It was bad, but it wasn’t as bad as I thought it would be.” Satisfaction with care was mostly due to implicit trust in their clinicians. Participant G-R-2 exemplified what many expressed, “I totally, thoroughly trust these guys. I mean I just feel like they dedicate so much of their life to learning all of this that I put my money on them and I’m sticking it there!” All participants except one, who received surgery, indicated they would undergo their selected treatment again if necessary.

**Table 2:** Representative quotes

Participant ID	Patient as person—treatment
B-R-2	I would go with radiation any time
D-R-2	[The treatment] was excellent! The people were very professional. Treated you with dignity and respect. And thoroughly explained things, how the procedure would go, and…for something you have to do it was a pretty pleasant experience. There was no stress or strain or nothing like that
M-R-2	The nurses did a lot to talk me through it and I wouldn’t have gotten through it otherwise. And they treated me so nice, but I was sure glad when it was over
E-S-2	They [the clinicians] were very responsive. If my nurse wasn’t available, if she was taking care of something or somebody else and I push the light, another nurse would come in and help… It was one of the better experiences I had for surgery. It was well explained, what was going to happen beforehand. I was allowed to go home after 10 days of the surgery, which was remarkable in my opinion. On other surgeries they just told me what they were gonna do and then let me like, ferment there for a while. The doctor didn’t come into see me on a daily basis like they did at [A] up there
Post-treatment	
K-S-2	[The shortness of breath] is getting better every day
B-R-2	I didn’t think nothing of it [coughing up blood] cause they said that was gonna happen so… but it was only like for 2, 3 days tops. Other than that… it was very tolerable
H-S-3	Well I have to be frugal. I run out of energy quite quickly and so… I can’t be as active and I’m not as strong as I used to be. It’s about what I expect
D-R-2	I would much prefer to take the radiation treatments and that way they’d done the best thing they could do for me. Better than to say, “Well, we’re not sure it’s cancer so we just won’t do nothing,” and then a year or 2 years down the road, then it keeps growing and then pretty soon it- that’s the end of ya’
E-S-2	… if anybody’s contemplating doing this surgery at [A] up there, I suggest that they do it. They’ll get great care and increase their life expectancy and their quality of life

Participant ID	Information exchange—knowledge
F-S-3	One of the problems is that I’m getting messages relayed through the nursing staff and through the, you know, nursing assistants and so on, and my questions aren’t being answered. I don’t know why
B-R-2	Now if they’ve already got my lung I was curious, can you tell me if they got it all on the lung or not?… Well I don’t know because I don’t know what the results are. I don’t know if it slowed it down or if it’s killed it or if it was off target. I don’t know, I don’t have any information yet
I-R-2	Having to have the statistics is nice, but it isn’t so that I can count on them as much as it is to sort of say, ‘ok well if this happens then I can do this and if this doesn’t happen then, you know…’ so it’s sort of like without the statistics I might be just saying, “Well there wouldn’t be any point in having the radiation because I’m gonna die anyway so why bother?” So as far as if they refuse to give me statistics I’d be kind of wondering why

Participant ID	Shared decision making
H-S-3	It falls back on the doctor if the patient doesn’t have the ability or the tools to research the situation
F-S-2	I still don’t see that I really had much of a choice. I learned a long time ago if you’re going to go to experts for their opinion, then listen to their opinion. There’s no point in going to them if you’re not going to listen to them… if you don’t have the knowledge and you go to someone who has the knowledge, listen to what they have to say! And then ask them why they believe that way and let that, you know, convince you. You either agree with them or don’t agree with them and if you don’t agree with them then you pursue the, you know, the question farther to find out, until you come to a meeting of the minds. I think that’s what happened in this case. It just seemed like the wisest decision, the wisest way to go
E-S-2	Well when Dr. [name] told me gonna have to… remove the whole lung… “What?!?” Couldn’t quite believe it but… it would increase my chances of survival by, well, twice as much as if I hadn’t had done it. So I made the decision all by myself…. God gave us freedom of choice so we need to exercise that
D-R-2	And they don’t really try to sway you one way or the other. They just tell you pretty much how it is and then they give you their opinion, and I look at things like this, is they know a whole lot more about this stuff than I do. And if they say that the odds would be better to do it than not do it, uh, they’re just not talking to hear themselves talk. They’re telling you with their experience and knowledge that this is the best thing for you. But the decision is ultimately yours. And I like that. They don’t uh, just say, “Well we’re gonna do this.” You know? They give you a choice and you can take it or leave it, but when you do, if it don’t turn out right then you don’t have anybody to blame but yourself. Don’t be blaming them
Trust	
I-R-2	I mean they just sort of tell you, “This is what you need to do and this is what we scheduled for you, is that going to work for you? And if not, we need to do something.” And so I basically said it was going to work for me cause I pretty much didn’t have anything else to do. [Laughter]… I really have a tremendous amount of respect for [my doctor]. I kind of believe [them]. I mean sometimes doctors make me a bit skeptical but [that doctor] I believe. If [that doctor] tells me I need something then I really believe that I need it
L-R-2	I trust my doctors, I’ve made my decision from what they told me, and I stood by their decisions they gave me pretty much
F-S-3	Oh I believe the doctor was correct. I’m sure that Dr. [2] would not tell me it wasn’t cancerous if it was

##### Post-treatment quality of life

Half of all participants reported increased dyspnea after treatment compared to baseline, regardless of treatment. Most participants felt fatigued 1 month after SBRT treatments which almost completely resolved at 1 year post-treatment. Three participants who underwent surgery had worse dyspnea at 1 month compared to baseline, but by 12 months only one had worse dyspnea compared to baseline while the others improved to pre-treatment levels. The two whose breathing had improved by 12 months were pleasantly surprised, since they reported it was logical to have increased dyspnea from baseline since they had part of their lung removed. Participant H-S-3 explained, “Obviously since I lost a quarter of my lungs that would tend to make me feel shortness of breath.” All participants stated they were told about these possibilities prior to treatment and most expected them.

#### Information exchange

##### Knowledge

Few participants reported having remaining questions about their treatment. However, when queried, several reported a lack of knowledge about how and when follow-up would occur, and had remaining questions about their treatment outcome. A number of participants who received SBRT were unsure about the status of their disease and questioned if they were “cured.” These participants described feeling anxious regarding their first surveillance scan. One participant (M-R-3) in particular stated, “And still to this day I’m unsure what my condition is. I don’t know whether it’s metastasized or if I’m in good shape.” Most communication deficits in both groups involved a lack of knowledge regarding the overall follow-up plan (Table 2).

#### Shared decision making

We asked several questions about the process of shared decision making (SDM) in general and the actual treatment decision. The interviewer(s) frequently had to explain what was meant by the decision making process and gave available options since participants often did not realize a decision was made. Two participants who received surgery felt they had no treatment choice. Participant K-S-2 said, “I wouldn’t say I had any other option other than the surgery,” but nonetheless remained satisfied with the decision making process. Remaining participants felt like they made the decision after getting all of the information they needed.

Almost all participants desired SDM, explaining the ideal decision making process as obtaining all of the facts and options from their clinicians before making their own decision. As participant L-R-2 stated, “I don’t think that would be shared at all [if one person doesn’t participate]. I mean, you’re not sharing if you’re not talking!” Several discussed the importance of both parties being involved in the decision process and that this occurred with their own decision. However, when queried more extensively, their decision making process often did not seem to meet their own definition of SDM. For example, at the baseline interview the participants who were surgical candidates reported clinicians infrequently discussed SBRT with them, which they confirmed at follow-up. Almost all later reported that all patients should hear about all treatment options despite the ability to tolerate surgery since information is a key part of SDM.

Even if SDM did not seem to happen, participants were still very satisfied with the decision making process. Trust in the clinician seemed to be important at explaining this apparent contradiction. Participant M-R-2 indicated, “Well that’s the only reason that I did it [treatment]. I assume that [my doctor] knew what he was doing. That’s all you can do, you know?” see Table 2 for related quotes.

#### Recommendations

Participant recommendations are in Table 3.

**Table 3:** Recommendations for clinicians

Prepare for visits (get to know the individual)
Give thorough explanations possibly through use of decision aids
Be patient
Personalize as much as possible
Have a good attitude
Address quality of life

One participant suggested giving patients a multiple choice or true/false quiz before treatment to ensure they know the risks and benefits

### Discussion

We found that overall, participants were satisfied with their treatment experience and QOL 1 month and 1 year post-treatment. They would almost always select their chosen treatment again. Most participants were very satisfied with the information they received, although many still had knowledge gaps about their treatment outcome and follow-up, especially those who underwent SBRT. Remaining questions and knowledge gaps were not clearly linked to reports of dissatisfaction, likely based on trust in their clinicians. Most participants felt they shared the treatment decision with their clinician. They believed all patients should be told about both treatments regardless of surgical candidacy although most did not feel they themselves received this information.

At the 1 month post-treatment visit, patients who received surgery indicated their QOL was still fairly high and not too different from baseline despite experiencing the expected side effects of surgery. Our findings suggest that future RCTs and observational trials will find worse short-term symptoms but similar QOL for patients undergoing surgery compared to those undergoing SBRT. The treatment experience was similar for both groups, which adds to the growing body of literature describing the possible equipoise between surgery and SBRT [5].

Participants in our study reported being well-informed about their treatments. Some, though, were unaware of their oncologic outcome (mostly those who underwent SBRT) and were distressed about their first surveillance scan. In other settings, distress is associated with lower adherence [12–15] so it will be important to evaluate if these patients are less likely to undergo surveillance imaging. The patient-centered communication model suggests that eliciting patients’ worries and concerns can mitigate distress [11]. Even when high quality care is not obtained, trust has been associated with self-reported high quality communication [16] and satisfaction with care [17] and high levels of trust are associated with improved adherence [18]. It is possible that trust as well as the positive treatment outcome are more essential than communication for patients with early stage lung cancer. The PCC model also points to other domains of communication that may be important to improving care, such as addressing patients’ values [11].

Participants did not describe key aspects of SDM like information gathering, discussing patient values and preferences, or developing a partnership [19]. These findings reflected results found in our qualitative study of clinicians, where clinicians confirmed they did not often discuss other treatments or ask about values and preferences directly [20]. Thus, it is not clear if SDM actually occurred had the decision been objectively recorded, despite reports that it did. Participants were still satisfied with the decision making process with little to no regret about their selected treatment. This seeming inconsistency may be due to the difficulty of measuring SDM. More and better quantitative tools are needed to improve consistency across studies [21]. It is important to note that some domains of communication may be associated with improvement in some outcomes but not others. For instance, our findings indicate some domains, like SDM, may be unnecessary for high quality care in certain clinical situations.

In order to address patient-reported communication gaps relating to treatment outcomes and follow-up procedures, clinicians and health care systems may need to develop improved processes or materials to ensure patients receive accurate information and ensure trust. Both treatment options should be discussed openly with all patients, despite their ability to tolerate surgery, explanations should be thorough, and personalized.

## Limitations

- We included participants from three health care settings in the Pacific Northwest so results may not be generalizable.
- Patient and clinician personal characteristics may influence perceptions of care and recall.
- We did not directly observe patient-clinician interactions so findings may not reflect actual practices, although the results are mirrored by our interviews with clinicians [20].
- Our results are subject to participant recall bias, as well as moderator acceptance bias [22].

## Abbreviations

HRQOL: health-related quality of life
NSCLC: non-small cell lung cancer
OHSU: Oregon Health & Science University
PCC: patient-centered communication
SBRT: stereotactic body radiotherapy
SDM: shared decision making
